# 

**Published:** 1910-04-30

**Authors:** 


### Tabes and Alcoholic Neuritis.

In certain cases the diagnosis between tabes dorsalis and alcoholic peripheral neuritis is decided by testing the degree of muscular power the patient still retains. In tabes there is no actual loss of strength in the legs, although from inco-ordination the patient may say he is too weak to walk; even a bed-ridden tabetic may have good muscle power in the legs, whereas peripheral neuritis causes absolute weakness.

### Acute Diseases of the Skin.

The imperative necessity of rest in acute diseases of the skin is too little insisted upon in general practice. The functions of the skin being interfered with, any exertion on the patient's part, or exposure to marked variations of temperature, can only still further impair those functions and retard recovery. By rest in bed much irritation is avoided and hyperæmia is reduced by the horizontal position, but above all the utmost benefit may be derived from the facility with which local remedies can be thoroughly applied, instead of in the usual half-hearted fashion.—*Dr. J. M. H. Macleod.*

### THE GRADUATES' MEDICAL SCHOOLS.

DIARY FOR THE WEEK, MAY 2 to MAY 7.

### CENTRAL LONDON THROAT AND EAR HOSPITAL, Gray's Inn Road, W.C.

At 3.45 p.m.—**May 3,** Dr. Atkinson, **Nose.**
3.45 p.m.—**May 6,** Dr. Dan McKenzie, **Labyrinth.**

### ROYAL SOCIETY OF MEDICINE, 15 Cavendish Square, W. (Temporary address during building.)

At 4.30 p.m.—**May 3, Therapeutical and Pharmacological Section:**

*Annual General Meeting.*—Dr. C. Bolton: "Some Points in the Treatment of Gastric Ulcer." Dr. Langdon Brown: "An Inquiry into the Value of Rectal Feeding."

(A meeting of the Council of the Section will be held at 4 p.m.)

At 5 p.m.—**May 6, Laryngological Section:**

At 11 Chandos Street, W.

*Cases*: Cases will be shown by Dr. Dundas Grant, Mr. Herbert Tilley, Mr. A. R. Tweedie, Dr. Watson Williams, Dr. G. C. Cathcart, and others.

At 10 a.m.—**May 7, Otological Section:**

At 11 Chandos Street, W.

*Paper*: Dr. Wyatt Wingrave: "Notes on the Pathology of Cholesteatoma."

*Cases and Specimens.*—Mr. A. L. Whitehead: Temporo-sphenoidal abscess; vomiting entirely absent. Dr. P. H. Abercrombie and Dr. Dan McKenzie: Hysterical deafness with active vestibular reactions. Dr. H. J. Davis: Deformity of both pinnæ resulting from perichondritis following double mastoid operation. Dr. Hunter Tod: (1) Exostosis of right external meatus. (2) A case in which the clinical symptoms simulated a cerebellar abscess. (3) Cholesteatoma or keratosis obturans of the external auditory canal. Mr. George Wilkinson: Two cases of cholesteatoma of unusual size. Dr. P. Watson Williams: Myelogenous sarcoma of the right temporal bone with extension through the external meatus, resembling an aural polypus. Mr. G. J. Jenkins: Fracture of the temporal bone.

ANNOUNCEMENTS.—General Meeting of Fellows: Friday, May 13, at 5 p.m.; Election of Fellows: The following candidates will be balloted for: Evelyn George Beadon Adams, M.B., F.R.C.S.; David Brown, M.D.; Archibald Montague Henry Gray, M.D., B.S., F.R.C.S.; Reginald Affleck Greeves, M.B., B.S., F.R.C.S.; Claud Maxwell Pennefather, M.B., B.S.; George French Stebbing, M.B., B.S., L.R.C.P., M.R.C.S.; Jane Elizabeth Waterston, M.D., L.&M.R.C.P.I., L.R.C.S.; William Henry Wynne, M.D., M.B., Ch.B., M.R.C.P.

Special Meeting of Fellows of the Society: May 18, at 5 p.m., at Morley Hall, George Street, Hanover Square, W. Sir Almroth Wright, F.R.S., will open a discussion on "Vaccine Therapy: its administration, value and limitations." The discussion will be continued on May 25, June 1, June 8, and June 15. The Council has arranged for a number of Fellows to speak on the above days. Any Fellow who wishes to take part in the discussion is requested to communicate with the hon. secretaries.

### NATIONAL HOSPITAL FOR THE PARALYSED AND EPILEPTIC, Queen Square, Bloomsbury, W.C.

At 3.30 p.m.—**May 3,** Dr. Risien Russell, **Paralysis of Cranial Nerves.**
**May 6,** Dr. Gordon Holmes, **Functional Localisation in the Brain.**

### LONDON SCHOOL OF CLINICAL MEDICINE, Seamen's Hospital, Greenwich, S.E.

At 3.30 p.m.—**May 4,** Mr. Cargill, **Sympathetic Ophthalmia.**
2.30 p.m.—**May 5,** Dr. Rankin, **Duodenal Ulcer.**
2.15 p.m.—**May 6,** Dr. Bradford, **Angina.**

### MEDICAL GRADUATES' COLLEGE AND POLYCLINIC, 22 Chenies Street, W.C.

At 4 p.m.—**May 2,** Dr. Graham Little, **Clinique (skin).**
5.15. p.m.—**May 2,** Dr. F. M. Sandwith, **Malta Fever.**
4 p.m.—**May 3,** Dr. T. N. Kelynack, **Clinique (medical).**
5.15 p.m.—**May 3,** Mr. Mackenzie Davidson, **Localisation by Means of X-rays.**
4 p.m.—**May 4,** Mr. D. C. L. Fitzwilliams, **Clinique (surgical).**
5.15 p.m.—**May 4,** Mr. Ernest Clarke, **Optic Atrophy (with lantern slides).**
4 p.m.—**May 5,** Sir Jonathan Hutchinson, **Museum Demonstration.**
5.15 p.m.—**May 5,** Dr. St. Clair Thomson, **The Operation of Laryngo-Fissure and its Indications.**
4 p.m.—**May 6,** Mr. J. Gay French, **Clinique (Ear, Nose, and Throat).**

### THE POST-GRADUATE COLLEGE, West London Hospital, Hammersmith, W.

At 10 a.m.—**May 2,** Demonstration by Surgical Registrar.
10 a.m.—**May 5,** Demonstration by Surgical Registrar.

### THE MEDICAL LIBRARY.

Post Free.

"Medical History: From the Earliest Times." By E. T. Withington, M.A., M.B. Oxon ... 12s. 6d.

"The Consumptive Working Man. What Can the Sanatoria Do for Him?" By Noel D. Bardswell, M.D. ... ... ... ... 10s. 10d.

"Photomicrography." By Edmund Spitta, M.R.C.S. With 41 Half-tone Reproductions from original negatives and other Illustrations ... ... 12s. 0d.

Of all booksellers or of The Scientific Press, Limited, 28 and 29 Southampton Street, Strand, London, W.C.

### Gifts and Bequests.

Mr. Pascoe Fenwick, of Kensington, W., has left £500 to the Gravesend Hospital.

The treasurers of the Middlesex Hospital have received a further £100 from Mr. C. H. Berners.

Miss Mary Rimmer, of Manor House, St. Bees, Cumberland, has left £100 to the Whitehaven and West Cumberland Infirmary.

The Chelsea Hospital for Women has received from the trustees of the Zunz Bequest £2,000 to name a ward the "Annie Zunz Ward."

Miss Wilhelmina Peckover, of Wisbech St. Peter, Cambridge, has left £200 to the North Cambridgeshire Cottage Hospital.

The Leicester Infirmary has received £100 from the late Mr. James Foster Burgess and £100 from the late Mrs. Mary Anne Jones, both of Leicester.

The Rev. Richard Norris Gandy, of Bournemouth, has left £200 to the Brompton Hospital for Consumption and £100 to the Kent and Canterbury Hospital.

Mrs. Louisa Meek, of Newcastle-on-Tyne, has left £100 each to the Fleming Memorial Hospital, the Newcastle Royal Infirmary, and the Newcastle Eye Infirmary.

The committee for the removal of King's College Hospital to South London has received £200 from the Clothworkers' Company (second instalment of a second £1,000).

Sir Walter Scott, Bart., has bequeathed £500 to the Fleming Memorial Hospital for Sick Children, Newcastle-on-Tyne; £1,000 to the Royal Victoria Infirmary, Newcastle.

Among the latest contributions received at the Bank of England for King Edward's Hospital Fund for London is an annual subscription of £500 from Messrs. N. M. Rothschild and Sons, and £500 from the Clothworkers' Company.

The Queen has sent her annual subscription of £10 to the Mary Wardell Convalescent Home for Scarlet Fever, of which she is the patroness, graciously expressing her appreciation of the value of the institution.

The donations and subscriptions at the recent festival banquet of the Finsbury Dispensary amounted to £631.

The theatricals organised recently by Florence Lady Clarke-Jervoise, produced on Tuesday night alone £300 for the East London Hospital for Children at Shadwell.

Under the will of the late Mr. Samuel Jaques, of Leicester, bequests of £100 are made to the infirmary and £300 to the Children's Hospital in connection therewith, both free of duty.

The Court of Common Council has made the following grants: To the Metropolitan Hospital, £150; the Royal Hospital for Incurables, £100; £50 each to the Elizabeth Fry Refuge, Miller Hospital, Greenwich.

Mrs. Lydia Matilda Gold, Brighton, widow of the gentleman who was murdered by Percy Lefroy in an express train from London to Brighton in 1881, has left £50 to the London Hospital, £50 to the Sussex County Hospital.

The Royal Hospital for Incurables, Putney Heath, has received £100 from the Goldsmiths' Company, ten guineas from the Pewterers' Company, and £10 10s. from Alderman and Colonel Samuel Wilson's Trust, on the nomination of Alderman Sir John Pound.

Miss Martha Elizabeth Gray, of London, has left £1,000 to Guy's Hospital for the endowment of a bed for males in the Queen Victoria Ward of the hospital, and a sum not exceeding £200 for the endowment of a bed in Newbury Hospital.

The Treasurer of St. Bartholomew's Hospital acknowledges the following further contributions to the Special Fund: The Guardian Assurance Company, Ltd., £105; Mr. Harry Symington, £52 10s.; Evans Sons Lescher & Webb Ltd., £10 10s.

The Treasurer of University College Hospital has received the following in response to the special appeal for £10,000 before June 1 next: The Mercers' Company, £210, and Mr. W. C. Alexander, Mr. Roger Beck, Mr. Hugo Müller, Mr. Lionel Lucas, Mr. Henry Lucas, the Clothworkers' Company, Lord Southampton, and Mrs. Mirrielees, £100 each.

### THE QUESTION BOX.

**SPECIAL NOTICE.—Questions of interest affecting the honorary medical staff and hospital administration in all departments are invited. When any point arises we hope our readers will formulate it in a question and so co-operate to make this column of real value and interest. In this way the experience of the best-managed hospitals should be made helpful to the whole hospital world. We shall welcome the contribution of both questions and answers.**

**N.B.—The publication of an answer in this column does not necessarily preclude the publication of subsequent answers to the same question, for a question may involve points which require to be threshed out and fully discussed. Answers, if published, will be paid for at scale rates.**

### OUT-PATIENT CASUALTY ADMITTANCES.

QUESTION: Should accidents admitted as in-patients from the casualty department be counted as casualties?

### A Metropolitan View.

BY A LONDON HOSPITAL SECRETARY.

WHEN the uniform system of accounts was revised in 1906, the committee appointed for this purpose realised that it was essential that the statistics published by the various hospitals should be prepared upon a uniform system of registration of patients, otherwise any comparison of the cost of the work performed by the different institutions was quite impracticable.

In these circumstances the problem of the best method of registering patients received serious consideration. The first question which had to be answered was "What is one out-patient?" The committee recognised the danger of framing definitions, but they also realised that any system of enumerating out-patients which admits of the same patient being counted more than once was unsatisfactory; they ultimately decided upon the following definition of an out-patient, viz. "One person attending continuously for the same ailment, for however long a period."

They anticipated that objections would be raised to this definition, and that to avoid counting an individual more than once, no matter how conscientious the attempt, was very difficult. For instance, take the case of a patient who attends the casualty department for an emergent illness or injury, receives first treatment, and is then referred to the out-patient department for further treatment, and later on may be admitted as an in-patient, any system which would prevent the registration of such a case more than once in any one year must be elaborate and therefore costly.

It must, however, be admitted that, despite the difficulties attending the registration of patients, any system which permits the undue multiplication of the numbers is undesirable, and therefore should be avoided; even if the case I have referred to cannot be always prevented, no patient ought to be re-entered in the registers as a new patient because he has been attending beyond a fixed period.

In those hospitals where the letter system is in operation it is possibly unavoidable that patients attending for a period, say, of one year shall be required to provide a fresh letter every two or three months, but it is certainly objectionable that such patients should be re-entered as new ones and therefore figure in the statistics of work done as four or six patients, as the case may be.

It was finally laid down in the uniform system of accounts, as revised and adopted by the three distributing funds in December 1906, as follows :—

"An out-patient is a person attending continuously for the same ailment, for however long a period between January 1 and December 31 inclusive."

"A casualty patient is either an in-patient or an out-patient. If taken in, he counts as an in-patient. If treated and sent home, he counts as an out-patient. Care must be taken that where a hospital, for the purposes of its own arrangements or statistics, retains a distinction between casualty and other patients, no patient is counted twice, as in that event the total number of in-patients or out-patients, as the case may be, would be affected."

In the face of these definite instructions it is evident that accident cases which have been admitted as in-patients, after being first treated in the casualty department, ought not to be included in the statistics as both casualty cases and as in-patients.

### A Liverpool View.

By MR. FRANCIS H. MOORE, General Superintendent and Secretary, Royal Infirmary, Liverpool.

BEING attached to a hospital which is practically a "free" one, I do not appreciate the necessity for any distinction between "casualty" cases and others which are admitted to the wards. The primary function of a hospital, surely, is to deal with "accidents" and "urgencies," and these should have precedence over semi-chronic, trivial, and those cases requiring what I may term "operations of convenience." No doubt various considerations interfere with this proper arrangement, particularly in hospitals with medical schools.

---

SEASIDE CONVALESCENT HOSPITAL, SEAFORD.—The acting editor of *Burdett's Hospitals and Charities*, 1910, desires us to correct, with an expression of his regret, a printer's error on page 613, where the name of the Secretary, F. Maitland, has been wrongly inserted instead of Major Rupert Stewart.

THE Lord Mayor has arranged for a meeting of City men in support of the fund for new chemical laboratories at University College, London, to be held at the Mansion House on Tuesday, May 10, at 4 P.M. The speakers will include Lord Rosebery (Chancellor of the University), Lord Avebury, and Sir Felix Schuster (treasurer of the college). The fund has received several subscriptions lately, including £500 each from Mr. Otto Beit and Mr. James Eccles.

---

### COMING EVENTS OF THE WEEK.

**Saturday, April 30.**—Annual National Conference of Workers among Poor Children opens. Reception by Sir John and Lady Kirk, 32 John Street, W.C., 6 to 8.

Annual Dinner, French Hospital, Hôtel Cecil, 7.30. French Ambassador in chair.

**Sunday, May 1.**—Visiting Day at all the London Hospitals.

**Monday, May 2.**—Annual National Conference of Workers among Poor Children, Small Queen's Hall, Langham Place, 11 to 7. Conference resumes at 3 p.m. at 38*b* Curzon Street, W. Delegates' Tea, Sunderland House, 5 p.m.

Sixty-sixth Annual Festival Ragged School Union and Shaftesbury Society, Queen's Hall, 7. Lord Northampton, K.G., in the chair.

**Tuesday, May 3.**—Demonstration of 2,700 children from Ragged Schools in Upper Street, Islington, 6.30 p.m.

**Friday, May 6.**—Annual Meeting Medical Mission Auxiliary, Queen's Hall, 7 p.m. Mr. Carless, F.R.C.S., in the chair.

**Saturday, May 7.**—Second Municipal Health Exhibition opens at Agricultural Hall, Islington, N.

### Appointments.

R. E. V. Hale, Esq., B.A., M.R.C.S., has been appointed anæsthetist to the Samaritan Free Hospital for Women.

The following appointments have been made at the Seamen's Hospital, Greenwich: Assistant physician: Mr. Chas. Singer, M.B., M.R.C.P.; medical superintendent: Mr. R. D. O'Leary, M.B., B.S.; house physicians: Mr. H. O. West, M.B., B.S., Mr. Frank Standish, M.B., B.S., M.R.C.S., L.R.C.P.; house surgeons: Mr. G. Guy Butler, M.B., B.C., Mr. A. T. McCaw, M.R.C.S., L.R.C.P.

Last week, at the rooms of the Liverpool General Brokers' Association, Ltd., Mr. William Adamson presided over an election for the appointment of an honorary surgeon for the Royal Southern Hospital. Four candidates were nominated, and Mr. G. C. E. Simpson, F.R.C.S.(Eng.), having retired, a ballot was taken for the remaining candidates. The result of the final ballot was the election by 24 votes to 12 of Mr. Douglas Douglas-Crawford, F.R.C.S.(Eng.), honorary surgeon to the Liverpool Stanley Hospital, and lecturer in clinical surgery and surgical anatomy in the Liverpool University.

### Jottings.

Mr. John Burns has promised to pay a visit to the Royal Hospital for Incurables, Putney Heath.

Princess Christian last Saturday distributed at King Edward VII. Hospital, Windsor, medallions and certificates to members of the local St. John Ambulance Association.

The collections on Hospital Sunday at Melbourne last year, in round numbers £7,400, were the highest since 1888, which was known as the "Boom year," when the sum was £9,357.

The Lord Mayor, accompanied by the Lady Mayoress and the Sheriffs, will open the new nurses' home at the City of London Lunatic Asylum at Stone, near Dartford, on Saturday afternoon, May 28.

It was stated at a recent meeting of the Governors of the Royal Portsmouth Hospital that the Hospital Ball had yielded a profit of £190, and a hearty vote of thanks was given to Sir William and Lady Dupree, the Mayor and Mayoress, for their kindness and trouble in the matter.

A scheme is on foot to establish a home for incurables in connection with the Birmingham and Midland Hospital for Women. It is proposed to begin with a nursing home near the hospital for six or eight beds. The cost of maintenance is estimated at £300, the friends of patients being expected to contribute a small sum weekly when able so to do. The home probably will be called "The Taylor Memorial Home," in recognition of the twenty-four years' service given to the hospital by the late Professor Taylor.

At the fortieth annual meeting of the North Wimbledon Hospital last Saturday it was stated that the King Edward Hospital Fund considered the present site (in Thurston Road) very suitable for extension, but, in view of the applications for building grants by it and also by the South Wimbledon and Merton Hospital, recommended their amalgamation. This has failed, so the North Wimbledon Committee will proceed with its original plan to enlarge, and the South Wimbledon Committee intends to build a new hospital with 40 beds.

Hospital Sunday will be on June 12.

The Princess of Wales has sent some flowers to the patients in the British Home and Hospital for Incurables, Streatham; and hampers of primroses to the Paddington Green Children's Hospital, and to the Royal Hospital for Incurables, Putney Heath, of which her Royal Highness is patron.

Waddilove's Samaritan Hospital for Women, the new Bradford institution given by Mr. J. K. Waddilove, has now been opened. One of its chief objects is to help those who are unable to pay the amount charged at nursing homes, but will gladly meet the low charges, from 12s. 6d. to £2 2s., it proposes.

The Lord Mayor and the Lady Mayoress, accompanied by the Sheriffs, visited in state last week the Royal National Orthopædic Hospital at Great Portland Street, W. Lord Denbigh, the hospital chairman, in welcoming the Lord Mayor, said that over £17,000 was still owing for building, and the interest on this was a great tax on the general fund. There were beds for 200 patients, but owing to want of money only 130 were occupied, while 400 patients were waiting. The hospital was the first-fruits of the policy of the King's Fund that special hospitals should as far as possible be amalgamated, and he appealed for increased support. The Lord Mayor then referred to the meeting to be held at the Mansion House on May 12 on behalf of the hospital.

### CONSUMPTION IN AUSTRALIA.

The treatment of consumptives in this new country is attracting widespread attention. The Melbourne Hospital in its sixty-first annual report shows that hopeless consumptives occupy its beds, and raise its death-rate disproportionately. With an increasing death-rate, the Government of Victoria has only provided a total of 202 beds for consumptives in the Austin Hospital for Incurables and the Macedon and Green Vale Sanatoria. In open-air camps consumptive patients, built up by good food, could live a strenuous open-air life under skilled supervision, and with fishing, gardening, fruit-growing, poultry-keeping, or dairy work become ultimately self-supporting. "In certain cases married consumptives could bring their families and be helped by the common dairy and garden. It would be an introduction to the most suitable life for all consumptive families."

THE HOSPITAL. April 30, 1910.

*Name* ..........................................

*Address* ..........................................

..........................................

This Coupon must accompany manuscript or contributions intended for The Hospital.

**The Institution Library.**

"Burdett's Hospitals and Charities," 1910. (Net, post free.) ... ... ... ... ... ... ...10s. 11d.

"Hospital Expenditure: The Commissariat." For the use of Managers. ... ... ... ... ... 2s. 6d.

"The District Nursing Association; Hints on how to Start it." (Net, post free.) ... ... ... 1s. 1d.

"A Legal Handbook for the use of Hospital Authorities." ... ... ... ... ... ... ... 2s. 6d.

Ledgers, Charts, Case-papers, and every kind of Institutional Literature.

Of all booksellers or of The Scientific Press, Limited, 28 and 29 Southampton Street, Strand, London, W.C.

